# Integrated remote sensing and petrological study of garnet-bearing rocks in the Arabian-Nubian shield: a case study from Wadi Shait-Wadi Gemal area, South Eastern Desert, Egypt

**DOI:** 10.1038/s41598-025-21601-6

**Published:** 2025-10-10

**Authors:** Mohamed A. Younis, Mahmoud H. Elyaseer, Taher M. Shahin, Hatem M. El-Desoky

**Affiliations:** 1 Consultants engineering office, Cairo, Egypt; 2 Geology Department, Faculty of Science, Al-Azhar University, PO Box 11884, Nasr City, Cairo, Egypt

**Keywords:** Garnet, ASTER, Landsat-9, Remote sensing, Hafafit–Nugrus, Arabian-Nubian shield, Egypt, Solid Earth sciences, Geology, Petrology

## Abstract

The Wadi Shait–Wadi Gemal district, within the Hafafit metamorphic dome of Egypt’s South Eastern Desert, represents a structurally complex segment of the Arabian Nubian Shield where garnet-bearing rocks are difficult to distinguish using conventional mapping. This study addresses this issue by integrating multi-sensor remote sensing (Landsat-9 and ASTER) with detailed field and petrographic investigations. Advanced image processing techniques, including false color composites (FCC), principal component analysis (PCA), minimum noise fraction (MNF), and proposed band ratios (BRs), were applied to enhance lithological discrimination and improve the detection of garnetiferous units. Field and petrographic analyses confirmed three principal garnet-bearing lithologies: garnet–muscovite–biotite schists, psammitic gneisses, and pegmatites. Garnet porphyroblasts were observed within quartz-mica matrices, reflecting a complex metamorphic history involving multiple deformation phases. The integration of remote sensing with petrological data allowed the production of a refined geological map and the precise delineation of garnet-enriched zones. This combined approach proved effective in resolving lithological complexities and significantly improves the understanding of garnet distribution in Precambrian metamorphic terranes.

## Introduction

Garnet is not only a key metamorphic indicator that reflects pressure-temperature conditions during orogenesis but also occurs in various magmatic environments. It is an economically valuable mineral, widely utilized as an abrasive, in water-jet cutting, and in industrial filtration processes. Additionally, it serves as a pathfinder mineral in mineral exploration^1^.

**Fig. 1:**
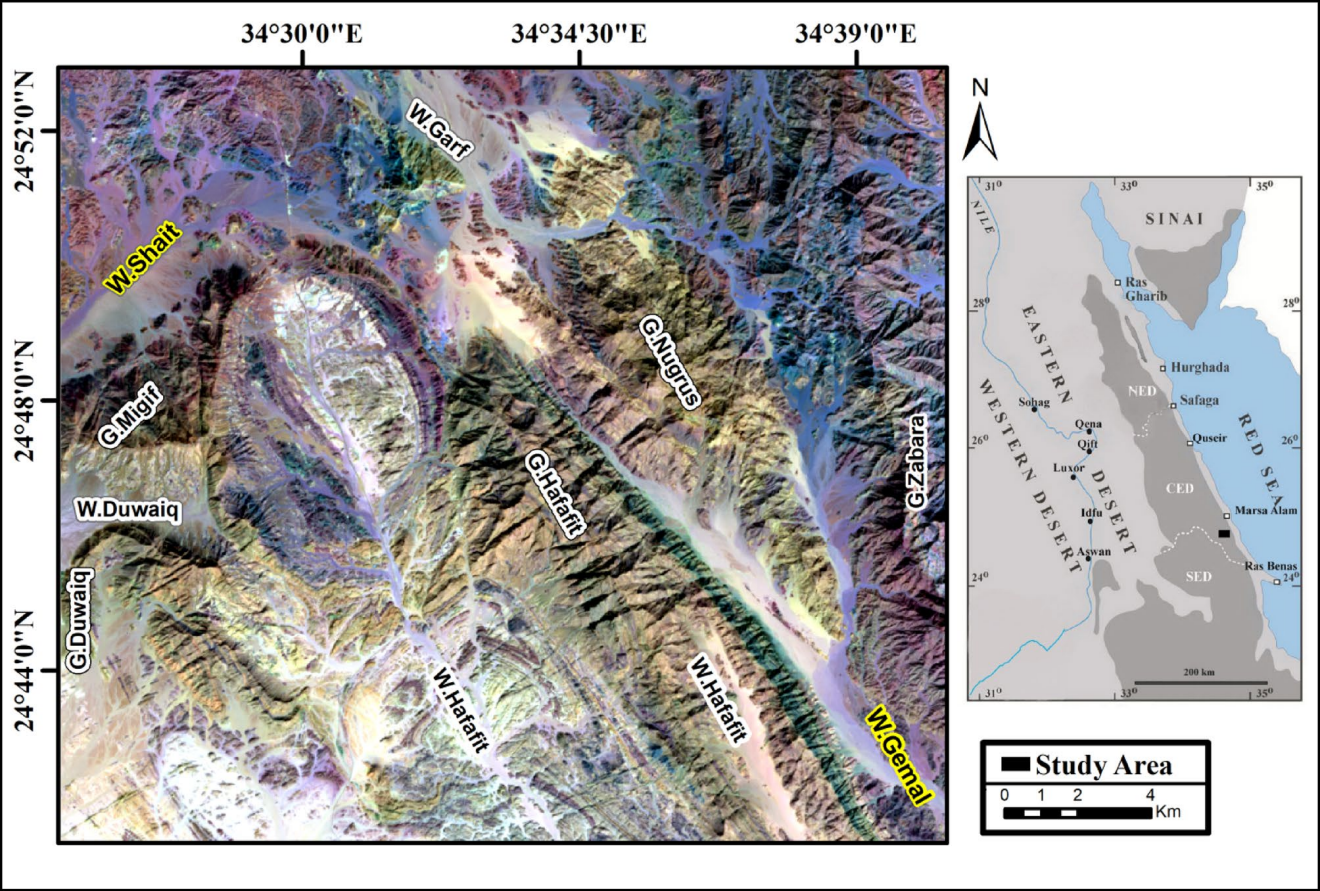
Landsat-9 image (7,5,3 in RGB) showing the location of the study area. (By ArcGIS v.10.5. https://www.esri.com/en-us/arcgis/products/arcgis-desktop/overview )

The Wadi Shait–Wadi Gemal district (WSGD) in Egypt’s Southeastern Desert contains a variety of garnet-rich metamorphic rocks that offer valuable information about the area’s Neoproterozoic tectonic and metamorphic history. A combination of fieldwork, petrography, and structural analysis highlights the intricate relationships between different rock units, particularly around major thrust faults and dome-shaped geological structures in the Hafafit metamorphic dome complex. The WSGD is a significant component of the Southeastern Desert Tectonic Zone (Migif-Hafafit belt), a crustal-scale Precambrian mobile belt extending NE-SW over the Eastern Desert Shield^2^ (Fig. 1). The WSGD dates to the Neoproterozoic era^2,3^ and consists of two primary litho-tectonic assemblages, divided by the Nugrus Thrust: the eastern Nugrus unit and the western Hafafit unit.

The geological framework of the Southeastern Desert is commonly interpreted using a three-part lithotectonic model. This model consists of thrust sheets of dismembered ophiolites, a metamorphosed assemblage of island arc volcanics and related sedimentary rocks, and later syn- to post-tectonic intrusive bodies (predominantly granites and gabbros). Together, these units record the region’s complex history of oceanic crust formation, volcanic arc activity, and continental collision.

This volcanic-sedimentary assemblage formed in a back-arc basin and is composed of light-colored gneisses, metabasites, and foliated metapelitic schists. The garnet–biotite gneisses in the Wadi Shait–Wadi Genal district crystallized in a later intraplate environment, reflecting a transition to post-collisional tectonic activity. In contrast, the biotite–hornblende gneisses originated during an earlier phase of arc accretion, indicating their formation in a convergent plate margin setting^4^.

In Egypt’s Eastern Desert, several granite plutons host abundant magmatic garnet. The Wadi Sikiat I-type granites contain ~ 4 vol% garnet (almandine-rich, 55–64 mol%), resembling those in Wadi El-Hima granites^5^. The Abu Had S-type granites and pegmatites have up to 10 vol% and 30 vol% garnet, respectively, with spessartine (32–48 mol%) and almandine (30–56 mol%) dominance^6^. Meanwhile, the Abu Diab A-type granites contain ≤ 2 vol% garnet, varying from spessartine (61–72 mol%) to almandine (25–35 mol%)^7^. These garnets are interpreted as products of peraluminous magmas in extensional or anorogenic regimes, consistent with their fractionated A-type granite hosts^7–9^ .

The WSGD was chosen for its uniquely exposed and undeveloped terrain, which offers a rare opportunity to study deep crustal processes that are typically hidden. As part of a major tectonic suture zone linked to the ancient formation of Gondwana, the WSGD preserves critical geological evidence of continental collision. Analyzing garnet minerals in this area can help accurately determine the temperature, pressure, and timing of these events. This localized study provides valuable global insights into tectonic theory, mountain-building processes, and mineral resource potential.

Remote sensing (RS) techniques have been extensively applied in the Arabian–Nubian Shield (ANS) and the Egyptian Eastern Desert for lithological mapping, hydrothermal alteration detection, and mineral exploration. For example, recent studies have demonstrated the effectiveness of multispectral and hyperspectral datasets such as ASTER, Landsat-9, Sentinel-2, and PRISMA in delineating lithological units and alteration zones associated with ore mineralization^10^. Additional investigations have highlighted the value of integrating RS with geological and geochemical analyses to resolve structurally complex terranes in the Eastern Desert and ANS, further reinforcing the importance of multi-sensor approaches in regional geological mapping^11,12^. Despite these advances, there remains a lack of systematic integration between RS analyses and petrological investigations specifically targeting garnet-bearing lithologies in the Wadi Shait–Wadi Gemal district (WSGD). This gap limits our understanding of the spatial distribution, textural characteristics, and petrogenetic significance of garnet within this structurally complex terrane. Therefore, this study represents the first attempt to integrate multi-sensor RS techniques (Landsat-9 and ASTER) with detailed field and petrographic analyses to detect, map, and classify garnet-bearing lithologies, ultimately producing an updated geological map for the WSGD.

This study integrates remote sensing techniques and petrological studies to detect, map, and classify garnet-bearing rocks within this structurally complex landscape. The application of multi-sensor satellite imaging Landsat-9 (OLI-2) and ASTER, along with advanced image processing techniques including FCC, PCA, MNF, and BR enhances lithological discrimination. These remote sensing results are supported by field-based observations and petrographic examinations of representative samples, aiming to refine the geological mapping and understanding of garnetiferous lithologies in the district.

## General geology

The Wadi Shait-Wadi Gemal district (WSGD) is located in the Southeastern Desert of Egypt, west of Mersa Alam city (Fig. 1). It forms a significant part of the Southeastern Desert Tectonic Zone, also known as the Migif-Hafafit Belt, which represents a NE-SW trending crustal-scale mobile belt within the Eastern Desert Shield^2^.

Geochronologically, this region is a Neoproterozoic age and structurally consists of two principal litho-tectonic units spilt by the Nugrus Thrust Fault: the eastern Nugrus unit and the western Hafafit unit^2,3^.

The low-grade metavolcanics and mica-schists that predominate in the Nugrus unit are frequently associated with metagabbros and altered ophiolitic ultramafic remnants, which are the remains of an oceanic arc-back arc system.

The Hafafit unit, in contrast, exhibits concentric zonation and contains banded amphibolites, interlayered biotite- and hornblende-gneisses, tonalitic to trondhjemitic granite gneisses, and psammitic gneisses that form the dome margins (Fig. 1). Amphibolite-metagabbro associations are also locally observed. In the Hafafit complex, five granite-cored domes are tectonically isolated from overlying low-grade schists and metavolcanics by shallow-dipping thrusts. These domes are diagnostic of metamorphic core complexes exposed during Pan-African deformation^13^. Both Nugrus and Hafafit units have been intruded by undeformed leucogranites, particularly along shear zones related to thrusting, indicating syn- to post-tectonic magmatism during crustal thickening and anatexis^3^.

**Fig. 2:**
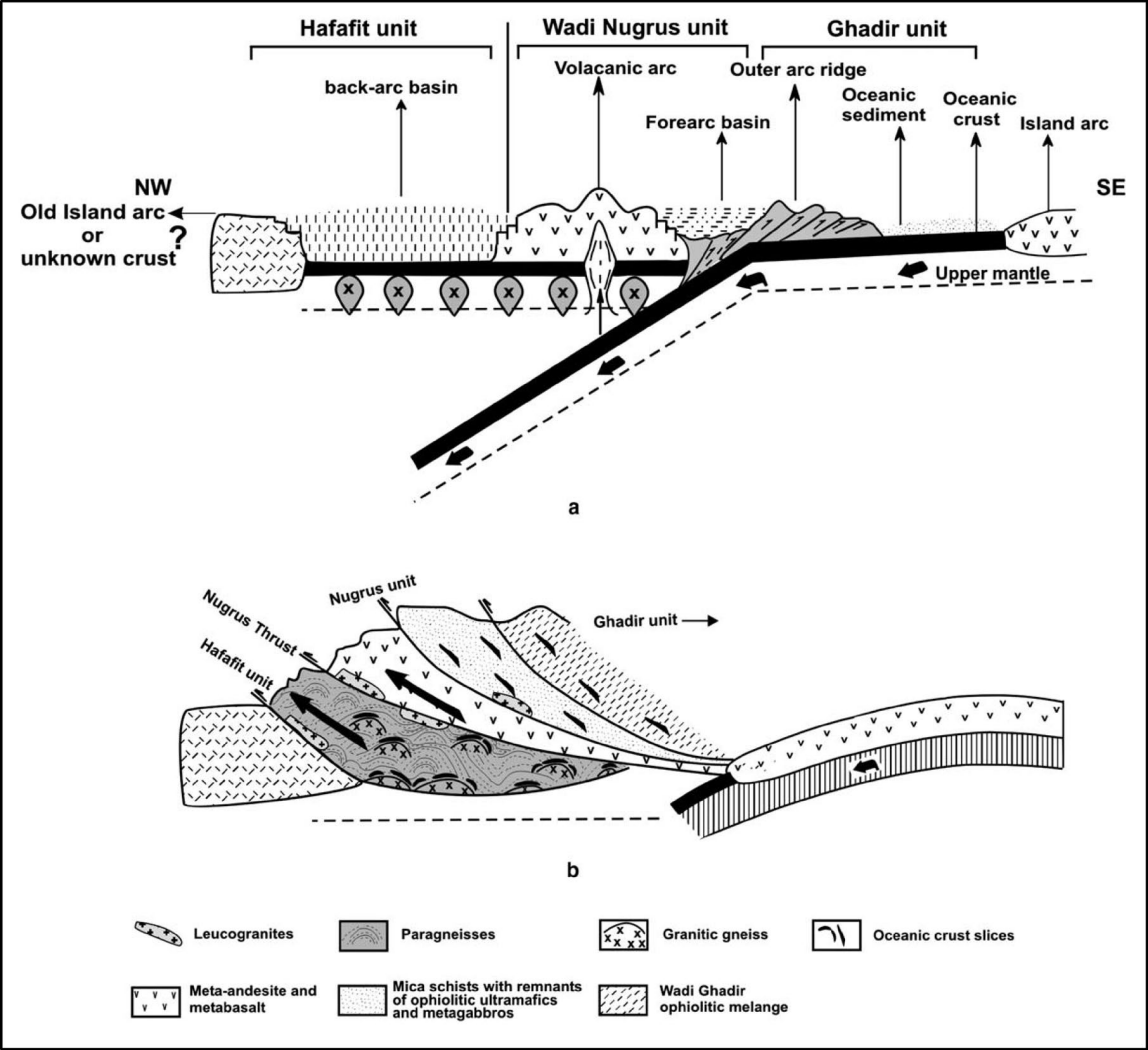
Schematic representation showing the tectonic evolution of Hafafit metamorphic complex and its relationship to the Nugrus unit. (**a**) Prior to 680 Ma, arc volcanism, arc-related plutonism, and the formation of back-arc basins in the Hafafit region were caused by the subduction of oceanic lithosphere, which also resulted in the detachment and pushing of ophiolites in Wadi Ghadir. (**b**) The collision and overthrusing of the Nugrus unit above the Hafafit complex^3^.

Regionally, the WSGD is linked to widespread Neoproterozoic deformation associated with the Pan-African orogeny, particularly involving NW-directed thrusting of the Nugrus unit over the Hafafit assemblage and the subsequent development of amphibolite-facies metamorphism (Fig. 2). The evolution of this region was further controlled by the Najd Fault System, which overprinted earlier accretion-related structures with large-scale NW-SE sinistral shearing and facilitated the exhumation of core complexes via orogen-parallel extension^14–16^.

Tectonic activity in the Hafafit-Ghadir area commenced before 680 Ma, marked by ophiolite emplacement and arc magmatism, followed by major crustal thickening around 600 Ma due to arc-continent convergence^3,17^. This tectonometamorphic history is documented in four deformation phases (D1-D4), ranging from early amphibolite deformation (D1), through arc-collision and shearing events (D2-D3), to later brittle reactivation during Red Sea rifting (D4). These phases collectively define the crustal evolution of the central Eastern Desert^18–20^.

**Fig. 3:**
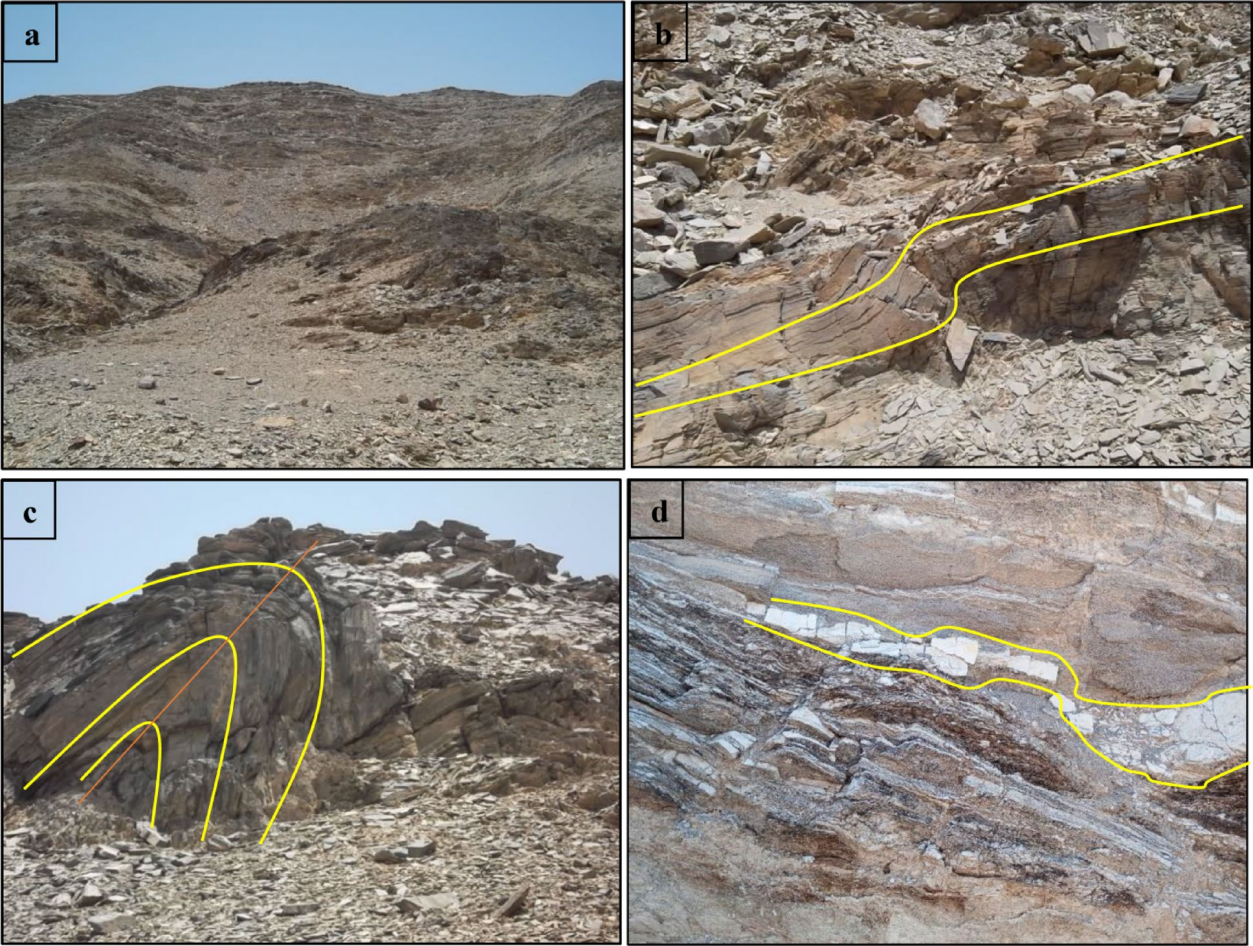
Field photo showing: (**a**) well-developed schistosity in volcano-sedimentary at wadi Abu Rusheid. (**b**) inclined foliated schists at Wadi Nugrus. (**c**) close-view highly folded schists at wadi Abu Rusheid. (**d**) boudinage pegmatite vein within passamitic gneiss at Wadi Hafafit.

The garnet mineralization in the Wadi Shait-Wadi Gemal and Wadi Hafafit areas of Egypt is closely tied to the tectonic and metamorphic processes of the East-African Orogeny. Subduction, continental collision, and intense metamorphism during the Neoproterozoic generated the heat, pressure, and aluminum-rich source rocks necessary for garnet formation. These processes created medium- to high-grade metamorphic terranes, later uplifted and eroded to expose garnet-bearing rocks. In Wadi Hafafit, garnet occurs in schists, gneisses, and pegmatite dikes, often along shear zones and near granite intrusions, highlighting the role of tectonic structures and magmatic activity in concentrating mineralization (Fig. 3a–d).

## Materials and methods

### Remote sensing data

**Fig. 4:**
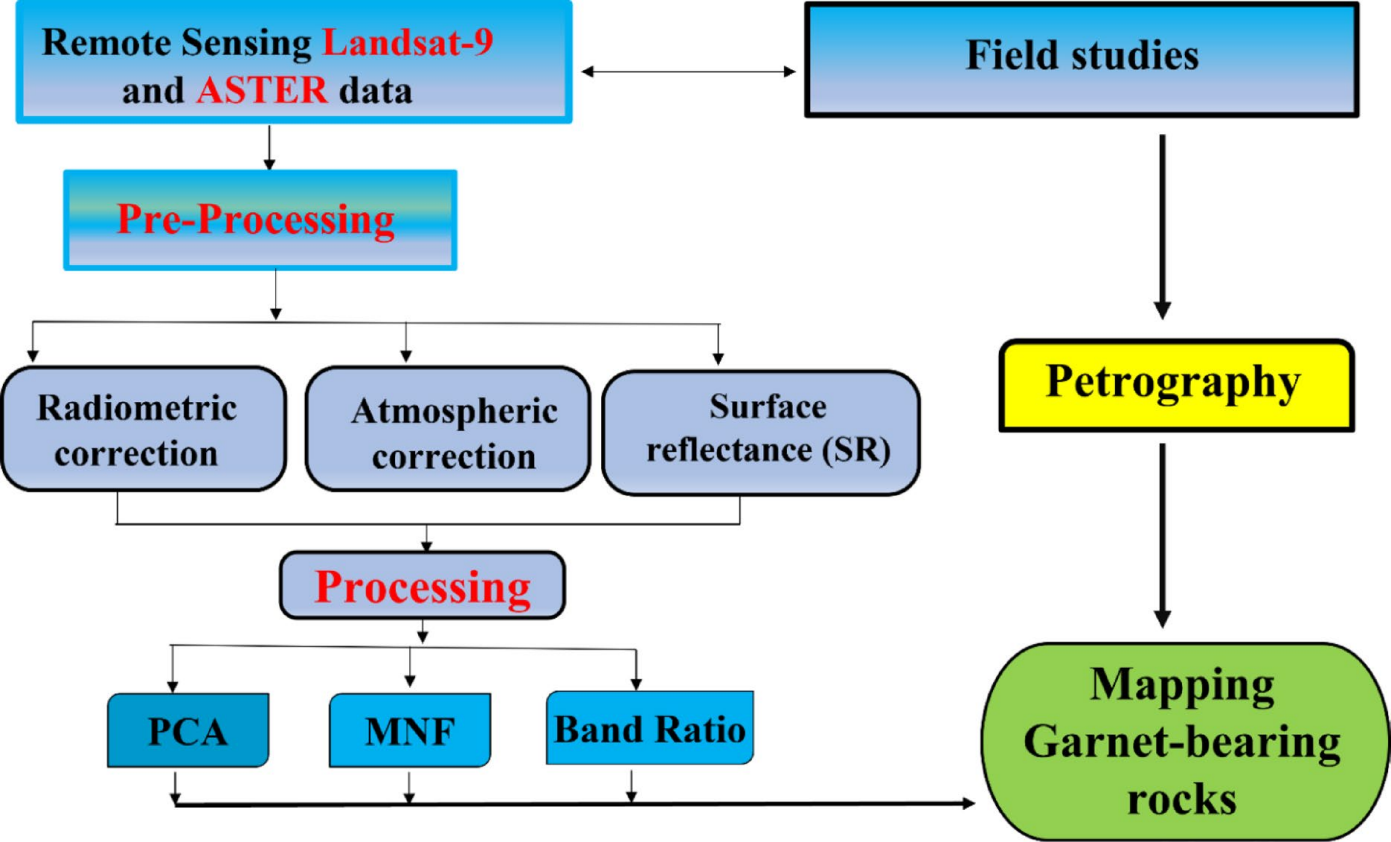
Overview of the flowchart methodological approach employed on the study area.

The present study employed Landsat-9 OLI-2 data and Advanced Spaceborne Thermal Emission and Reflection Radiometer (ASTER) images to examine the lithological rock units of the study area. Landsat-9 and ASTER data were pre-processed (radiometric, atmospheric corrections, and surface reflectance calibration) and analyzed using PCA, MNF, and Band Ratios, integrated with fieldwork and petrography to map garnet-bearing rocks (Fig. 4). Landsat-9 is equipped with two sensors: the Operational Land Imager-2 (OLI-2), which includes nine bands, and the Thermal Infrared Sensor-2 (TIRS-2), which offers two thermal bands (Table 1). The relevant OLI-2 scene (Path 173/Row 43) was obtained on October 29, 2023, from the USGS Earth Explorer (https://earthexplorer.usgs.gov/). ASTER data, acquired on October 30, 2003, were obtained from the NASA Earth data portal (https://earthdata.nasa.gov/). All datasets were registered to UTM Zone 36 N coordinate system based on WGS 84 datum.

**Table 1:** Radiometric properties of the optical and microwave data.

Sensor	Bands	Spectral region	Wavelength (µm)	Resolution (m)	Swath width (km)
ASTER	Band 1	VNIR	0.52–0.60	15	60
	Band 2		0.63–0.69		
	Band 3		0.78–0.86		
	Band 4	SWIR	1.60–1.70	30	60
	Band 5		2.145–2.185		
	Band 6		2.185–2.225		
	Band 7		2.235–2.285		
	Band 8		2.295–2.365		
	Band 9		2.360–2.430		
	Band 10	TIR	8.125–8.475	90	60
	Band 11		8.475–8.825		
	Band 12		8.925–9.275		
	Band 13		10.25–10.95		
	Band 14		10.95–11.65		
Landsat-9	Band 1	Coastal	0.433–0.453	30	185
	Band 2	Blue	0.450–0.515		
	Band 3	Green	0.525-0.600		
	Band 4	Red	0.630–0.680		
	Band 5	NIR	0.845–0.885		
	Band 6	SWIR	1.560–1.660		
	Band 7		2.100–2.300		
	Band 8	Panchromatic	0.500–0.680	15	185
	Band 9	Cirrus	1.360–1.390	30	
	Band 10	TIR	10.60-11.19	100	
	Band 11		10.60-11.19		

The preprocessing stage involved correcting for atmospheric effects using the FLAASH algorithm to retrieve accurate surface reflectance^12^, followed by spatial subsetting to align with the study area’s extent. All image processing procedures were executed using ENVI 5.6 software. The ILWIS program was employed to generate optimal False Color Composite (FCC) images for the study area, enhancing the visual discrimination of geological features. The analytical techniques employed comprised false color composites (FCC), principal component analysis (PCA), minimum noise fraction (MNF), and band ratios (BR), which collectively enabled the differentiation of lithological units, especially garnet-bearing rocks.

### Field and petrography

**Fig. 5:**
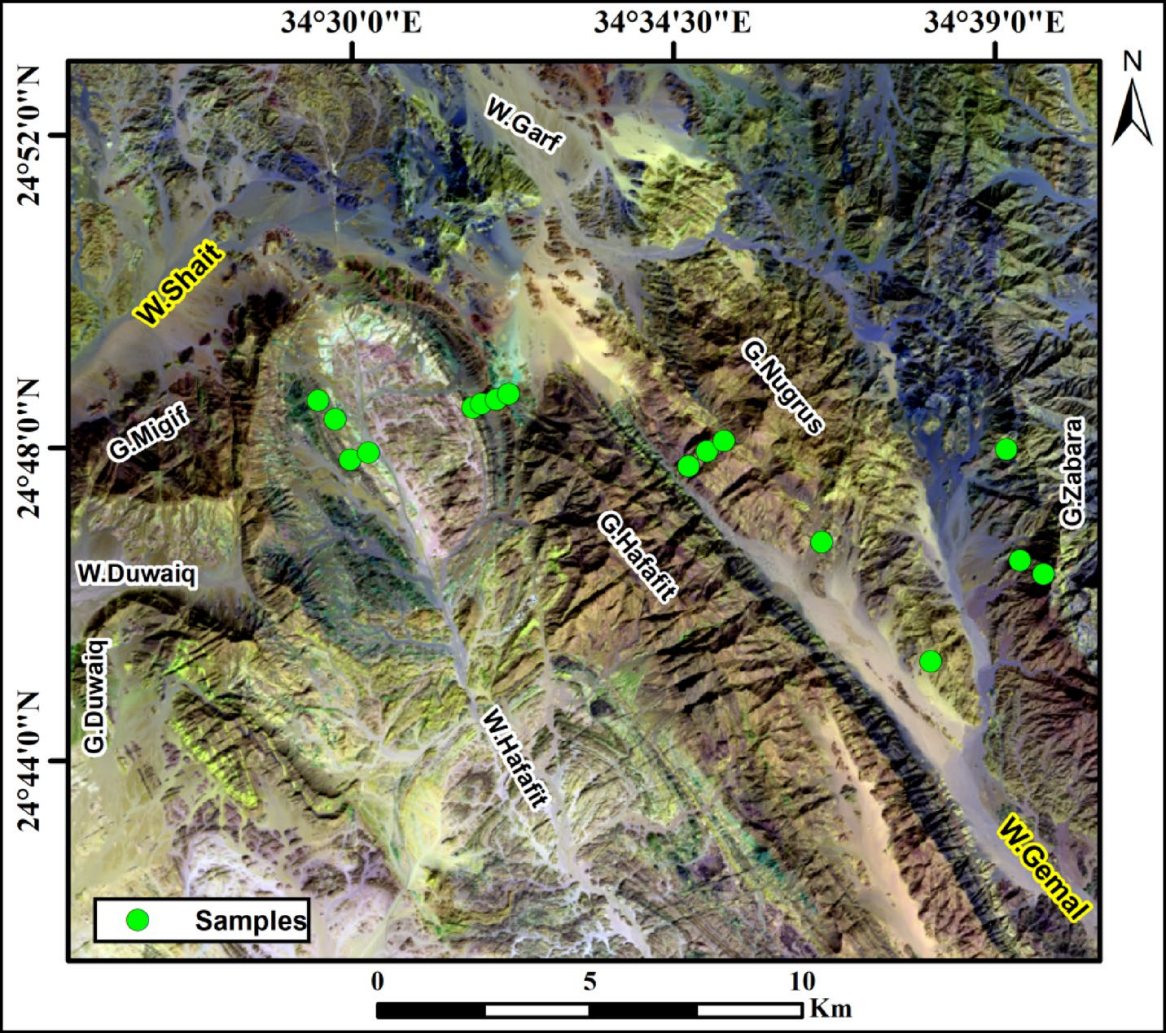
Landsat-9 image (7,5,1in RGB) showing the location of the collected samples from the study area. (By ArcGIS v.10.5. https://www.esri.com/en-us/arcgis/products/arcgis-desktop/overview )

More than thirty rock samples were collected from garnet-bearing lithological units within the delineated area (Fig. 5). Sixteen selected samples were cut into standard thin sections for petrographic analysis. The preliminary mineralogical composition of these materials was determined using a polarizing microscope at the Rock Preparation and Microscopy Laboratory, Geology Department, Al-Azhar University.

## Results

In this section, the results obtained from remote sensing analyses are integrated with field observations and petrographic descriptions. This combined approach allows for a clearer identification of high-potential garnet-bearing lithologies, as remote sensing anomalies are cross-validated with field evidence and thin-section analyses. Such integration ensures that the datasets complement each other, reducing uncertainties and providing a more accurate understanding of the spatial distribution and petrogenesis of garnet in the study area.

### Remote sensing processing

The garnet-bearing rocks were identified using a various of remote sensing methods, including false color composites (FCC), principal component analysis (PCA), minimum noise fraction (MNF) and band ratio (BR).

#### False color composite (FCC)

A color composite image can be generated using a combination of only three spectral bands. It is essential to select the most informative bands, minimizing redundancy and the number of highly correlated bands. The band combination that best highlight the target of interest are generally the most effective^21^.

**Fig. 6:**
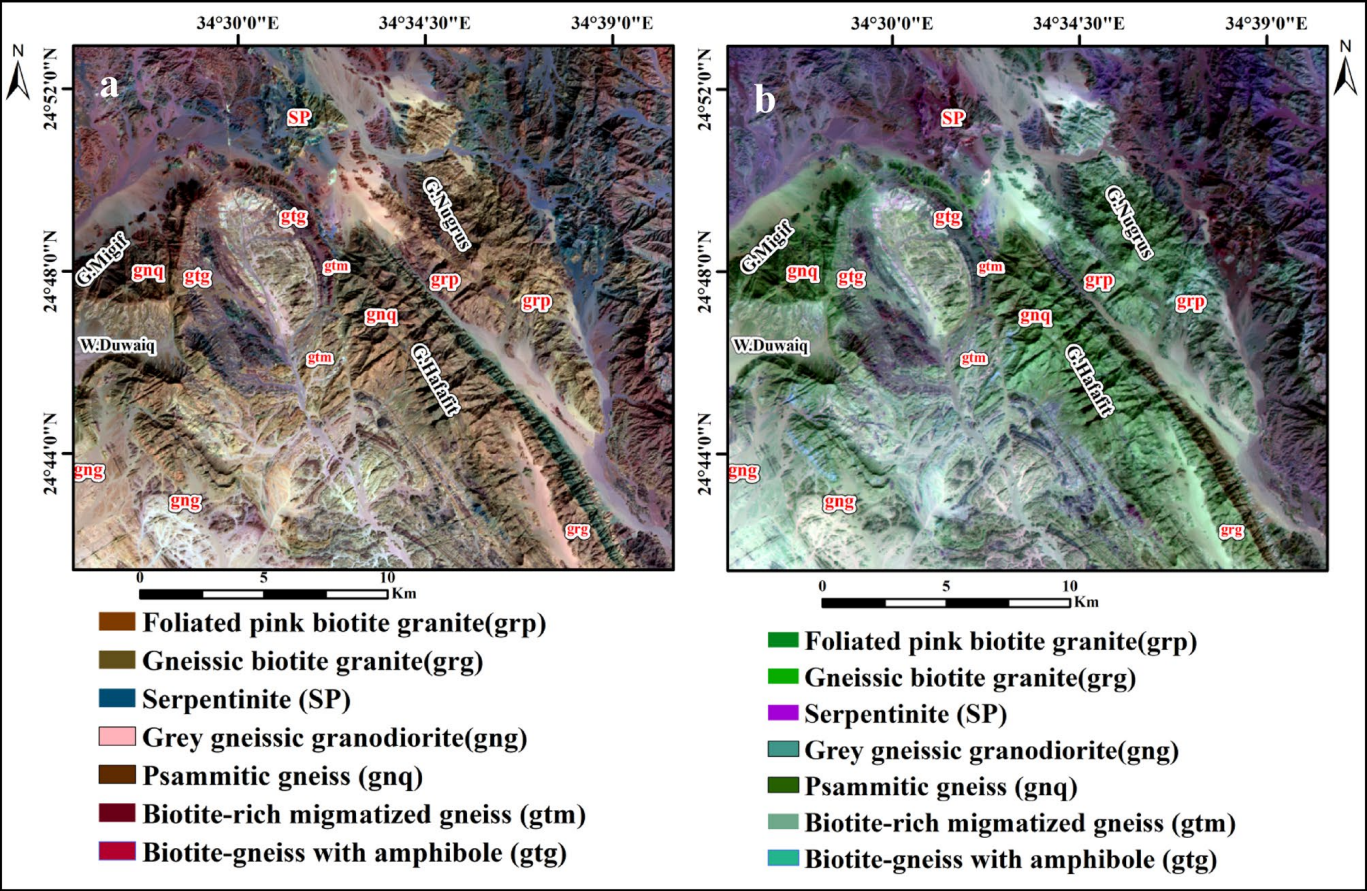
(**a**) Landsat-9 FCC (7,5,3 in RGB). (**b**) ASTER FCC (9.5.1 in RGB). (By ENVI version 5.6. https://www.l3harrisgeospatial.com/Software-Technology/ENVI ).

To generate the most effective false color composite (FCC) images for lithological discrimination within the study area, various spectral bands from both OLI-2 and ASTER datasets were systematically evaluated. This assessment aimed to identify the optimal band combinations that enhance the visual differentiation of lithological units, thereby supporting more accurate geological interpretations. Using the ILWIS program, the RGB color composite was blended with the Optimum Index Factor (OIF) approach. The OIF result analysis indicated distinct composites of OLI-2 and ASTER bands for enhancing the different lithological rock units in the present area (Table 2). These FCCs are derived from the relationship between the data’s correlation coefficients and standard deviations^22^. Several lithological units of the study area can be clearly distinguished by the Landsat-9 (7,5,3) RGB and ASTER (9,5,1) RGB data. In the Landsat-9 (7,5,3) RGB composite, the gneissic granodiorite is characterized by a pink hue, whereas the psammitic gneiss appears dark brown, and the biotite-rich gneiss is represented by a brown tone (Fig. 6a). The ASTER (9,5,1) RGB composite highlights the serpentinite in purple, the psammitic gneiss in dark green, and the biotite-rich gneiss in light green (Fig. 6b).

**Table 2:** Band combination with the highest OIF values.

Rank	OLI-2 Band triplet	ASTER Band triplet
1	753	951
2	751	961
3	761	971

#### Principal component analysis (PCA)

**Fig. 7:**
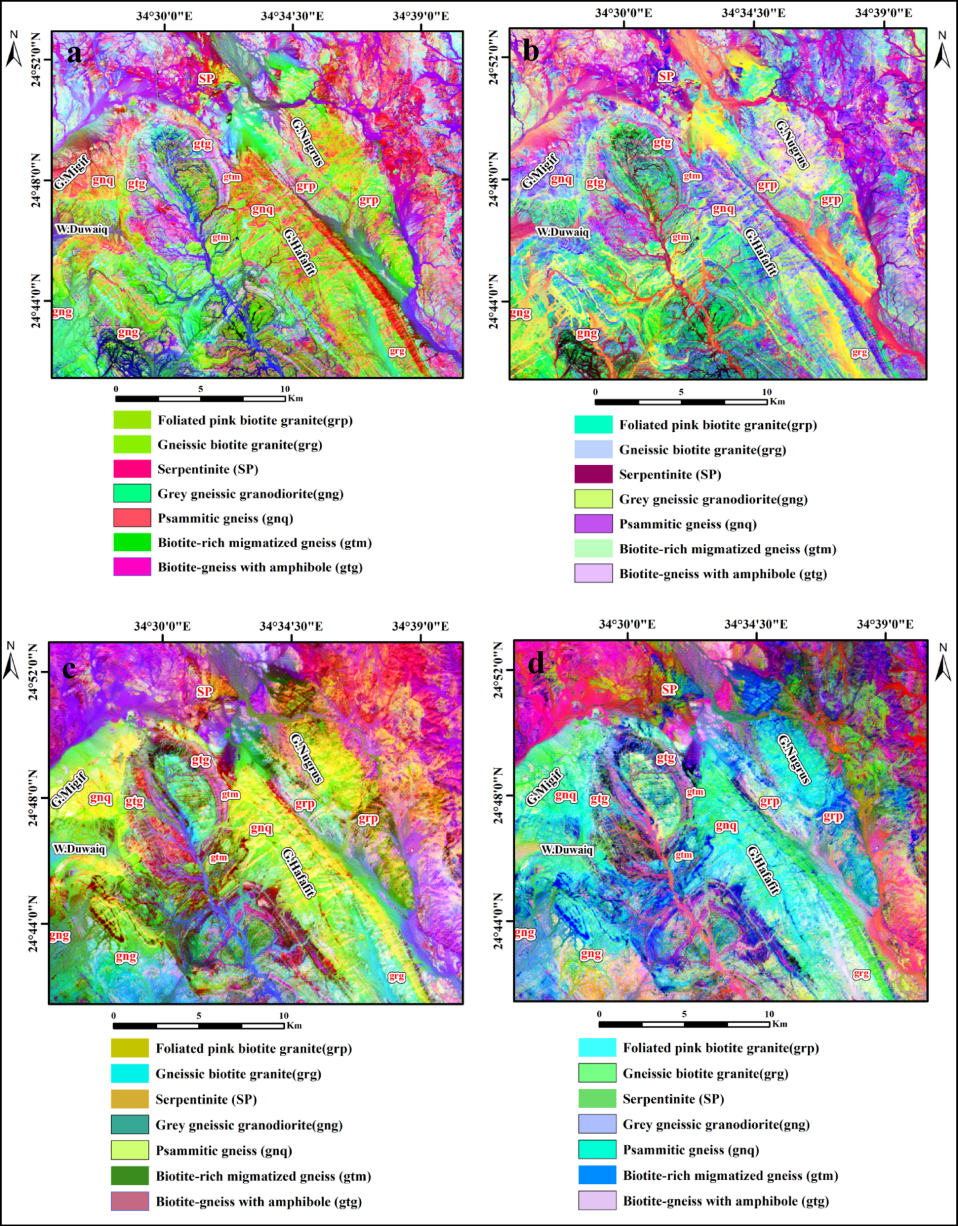
Principal component analysis band composites. (**a**) Landsat-9 (PC1, PC2 and PC3) RGB. (**b**) Landsat-9 (R: PC5, G: PC2, B: PC1) RGB. (**c**) ASTER (PC1, PC3, PC4) RGB. (**d**) ASTER (PC4, PC3, PC2) RGB. (By ENVI version 5.6. https://www.l3harrisgeospatial.com/Software-Technology/ENVI).

Principal component analysis (PCA) is a crucial statistical technique that constructs output bands that are statistically independent, facilitating the effective isolation of noise elements and minimizing the spectral dimensionality of the data^23^. PCA is an essential method for distinguishing between different rock types^24^. To enhance the interpretation of the distinct lithological units across the study area, particularly the garnet-bearing formations, PCA was applied to the OLI-2 and ASTER bands. The most significant bands of PCA results are PC1, PC2, PC3, and PC4, according to the eigenvalues derived from both datasets^25–27^. For the OLI-2 data, the first principal component (PC1) accounted for the largest variance (96.40%), while the second principal component (PC2) explained 2.44% of the variance. The final PCA bands were dominated by noise, high correlation, and the least variance. Based on the eigenvectors of the OLI-2 bands, PC1, PC2, PC3, and PC5 were selected as the most effective components (Table 3). A false color composite (PC1, PC2, PC3; RGB) highlights biotite schist in reddish-orange and biotite-rich gneiss in light mauve (Fig. 7a). Another composite (PC5, PC2, PC1; RGB) shows biotite schist in light blue and psammitic gneiss in sky blue (Fig. 7b). For ASTER data, PC1, PC2, PC3, and PC4 were the most informative. The composite (PC1, PC3, PC4; RGB) distinguishes gneissic granodiorite in dark turquoise and metagabbro metabasalt in dark mauve pixel color (Fig. 7c), while (PC4, PC3, PC2; RGB) highlights pegmatite bodies in blue and biotite schist in light green (Fig. 7d).

**Table 3:** Eigenvector matrix and loadings of principal component analysis (PCA) on landsat-9 images.

	PC 1	PC 2	PC 3	PC 4	PC 5	PC 6	PC 7
Band 1	-0.94170	-0.32103	0.09485	-0.01849	-0.02782	-0.00108	-0.00476
Band 2	0.24145	-0.84451	-0.45793	-0.10892	0.08302	0.00647	0.00090
Band 3	0.20250	-0.34696	0.84898	-0.26582	0.21518	0.00409	0.02947
Band 4	-0.05334	0.17872	-0.16485	-0.95084	-0.18250	-0.01088	-0.02274
Band 5	-0.10342	0.17392	-0.17947	-0.11301	0.91483	0.18942	0.20321
Band 6	0.01662	-0.03269	0.02497	0.01515	-0.27058	0.49285	0.82564
Band 7	-0.00873	0.00984	-0.02296	-0.00632	0.05106	-0.84914	0.52499
Eigenvalues	0.01824	0.00046	0.00015	0.00006	0.00001	0.00000	0.00000
	0.96401	0.02442	0.00766	0.00328	0.00048	0.00011	0.00005
%	96.40	2.44	0.77	0.33	0.05	0.01	0.01

#### Minimum noise fraction (MNF)

**Fig. 8:**
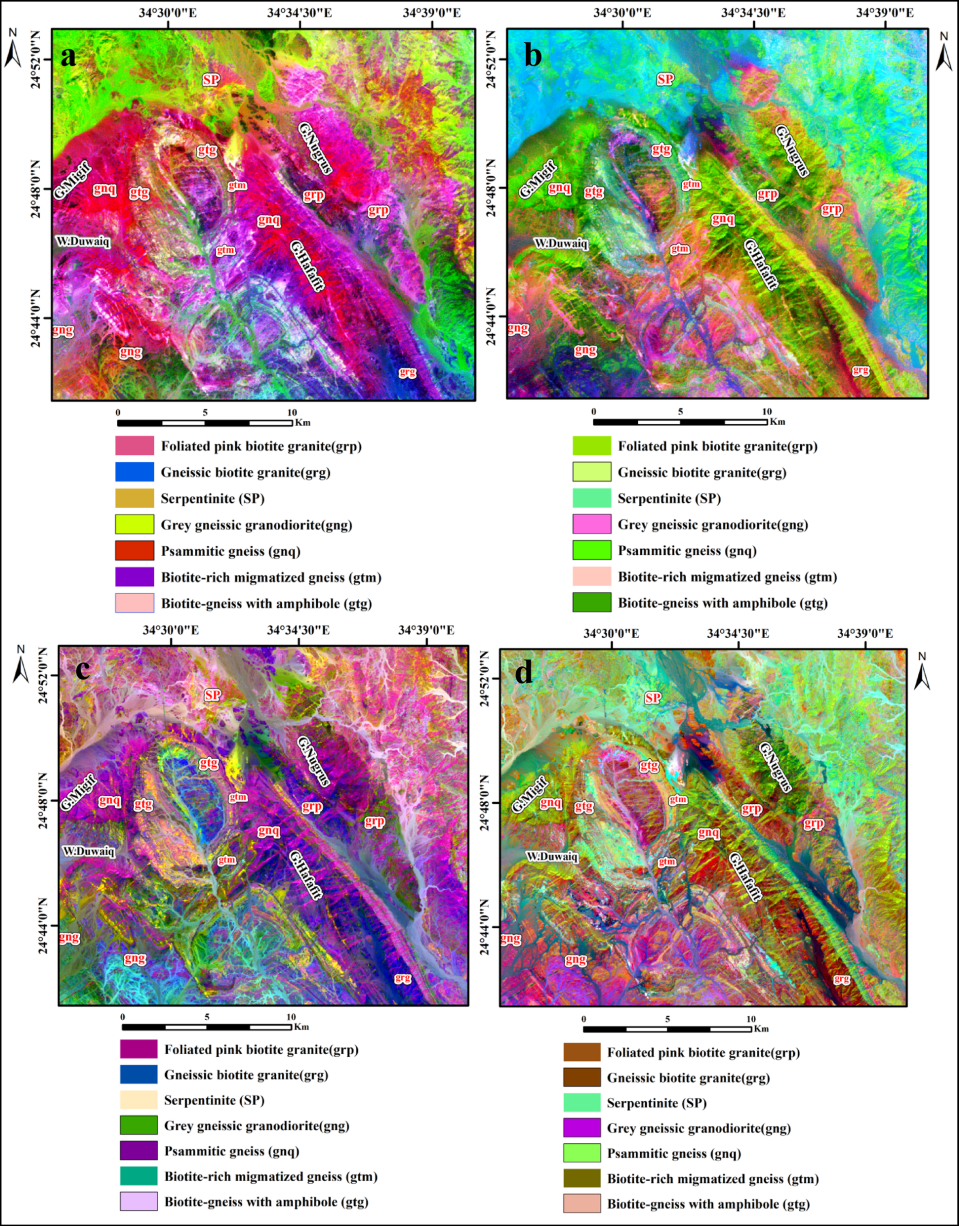
The minimum noise fraction (MNF) band composite. (**a**) ASTER (MNF3, MNF2, MNF4 in RGB). (**b**) ASTER (MNF 4, MNF 1, MNF 2 in RGB). (**c**) Landsat-9 (MNF 1, MNF 2, MNF 3 in RGB). (**d**) Landsat-9 (MNF 5, MNF 1, MNF 2 in RGB). (By ENVI version 5.6. https://www.l3harrisgeospatial.com/Software-Technology/ENVI).

The MNF transformation is an enhanced version of the PCA method, designed specifically to suppress image noise in selected spectral bands. The MNF technique is particularly advantageous when dealing with hyperspectral datasets, where signal-to-noise ratios may vary significantly due to fluctuations in signal strength across different spectral bands. This technique functions similarly to principal component analysis by first removing residual noise from spectral datasets to enable the identification of representative spectra. The MNF transformation proficiently differentiates between spectral bands that contain significant data related to the dataset’s overall variance and those predominantly influenced by noise^28,29^. In the present work, the minimum noise fraction transformation was employed to analyze datasets acquired from both OLI-2 and ASTER sensors. The results reveal that, in the case of ASTER image, the RGB combinations MNF3, MNF2, and MNF4 effectively highlighted the psammitic gneiss in cyan blue and the gneissic biotite granite in olive green (Fig. 8a). Additionally, the combination MNF4, MNF1, and MNF2 successfully depicted biotite schist in yellowish green and psammitic gneiss in olive green (Fig. 8b). For OLI-2 bands, the most informative band combinations were MNF1, MNF2, and MNF3, with psammitic gneiss appearing in dark purple (Fig. 8c), and MNF5, MNF1, and MNF2, which revealed gneissic granodiorite in dark purple and biotite-rich gneiss in cyan green (Fig. 8d).

#### Band ratio (BR)

Band ratioing is a widely used and effective technique in remote sensing image analysis, as it reduces the effects of illumination differences and enhances color contrasts among surface materials, thereby improving spectral anomaly detection^25^. The method involves dividing the Digital Number (DN) value of one spectral band by that of another, exploiting differences in spectral reflectance^30^. Various band ratio combinations from ASTER and Landsat-9 datasets have been frequently applied in geological studies to map lithological units and structural lineaments^11,31,32^, owing to their proven ability to highlight lithological discontinuities and lineaments^33,34^. This approach effectively emphasizes variations in spectral reflectance slopes between selected band pairs, regardless of the absolute reflectance values^35^.

**Fig. 9:**
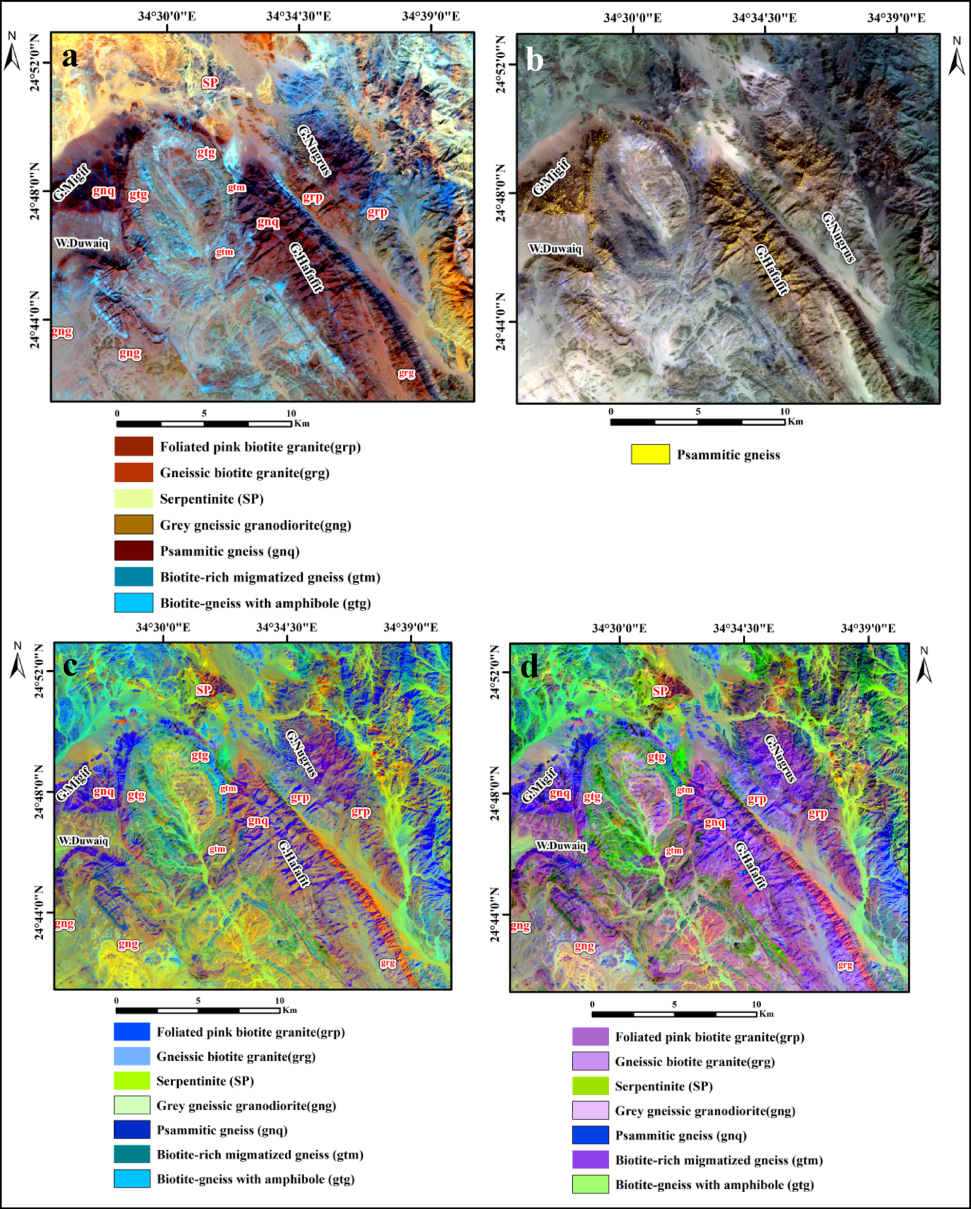
Band ratio composites discriminating garnet-bearing rocks. (**a**) ASTER band ratio composite image (b3/b4, b1/b5, b1/b3) RGB. (**b**) ASTER band ratio image (b8/b7) showing psammitic gneiss with yellow color pixel. (**c**) landsat-9 band ratio image (b5/b6, b2/b4, b5/b7) RGB. (**d**) landsat-9 band ratio image (b5/b6, b3/b5, b7/b6) RGB. (By ENVI version 5.6. https://www.l3harrisgeospatial.com/Software-Technology/ENVI).

In this study, band ratio combinations from ASTER and Landsat-9 data were applied to enhance the discrimination of garnet-bearing lithologies in the Wadi Shait–Wadi Gemal region. A novel ASTER composite (b3/b4, b1/b5, b1/b3 in RGB) successfully highlighted psammitic gneiss as dark red pixels (Fig. 9a), while the single-band ratio (b8/b7) further distinguished psammitic gneiss in yellow pixels, mainly concentrated in the central area (Fig. 9b). Complementary results from Landsat-9 data included a composite using ratios 5/6 (R), 2/4 (G), and 5/7 (B), where psammitic gneiss appeared violet-blue, biotite-rich gneiss cyan-blue, and gneissic granodiorite yellowish-blue (Fig. 9c). Another Landsat-9 composite (5/6, 3/5, 7/6 in RGB) enhanced garnet-bearing units, showing psammitic gneiss in violet-blue, biotite-rich gneiss in bluish-green, and gneissic granodiorite in violet-cyan (Fig. 9d).

#### Geological mapping results

**Fig. 10:**
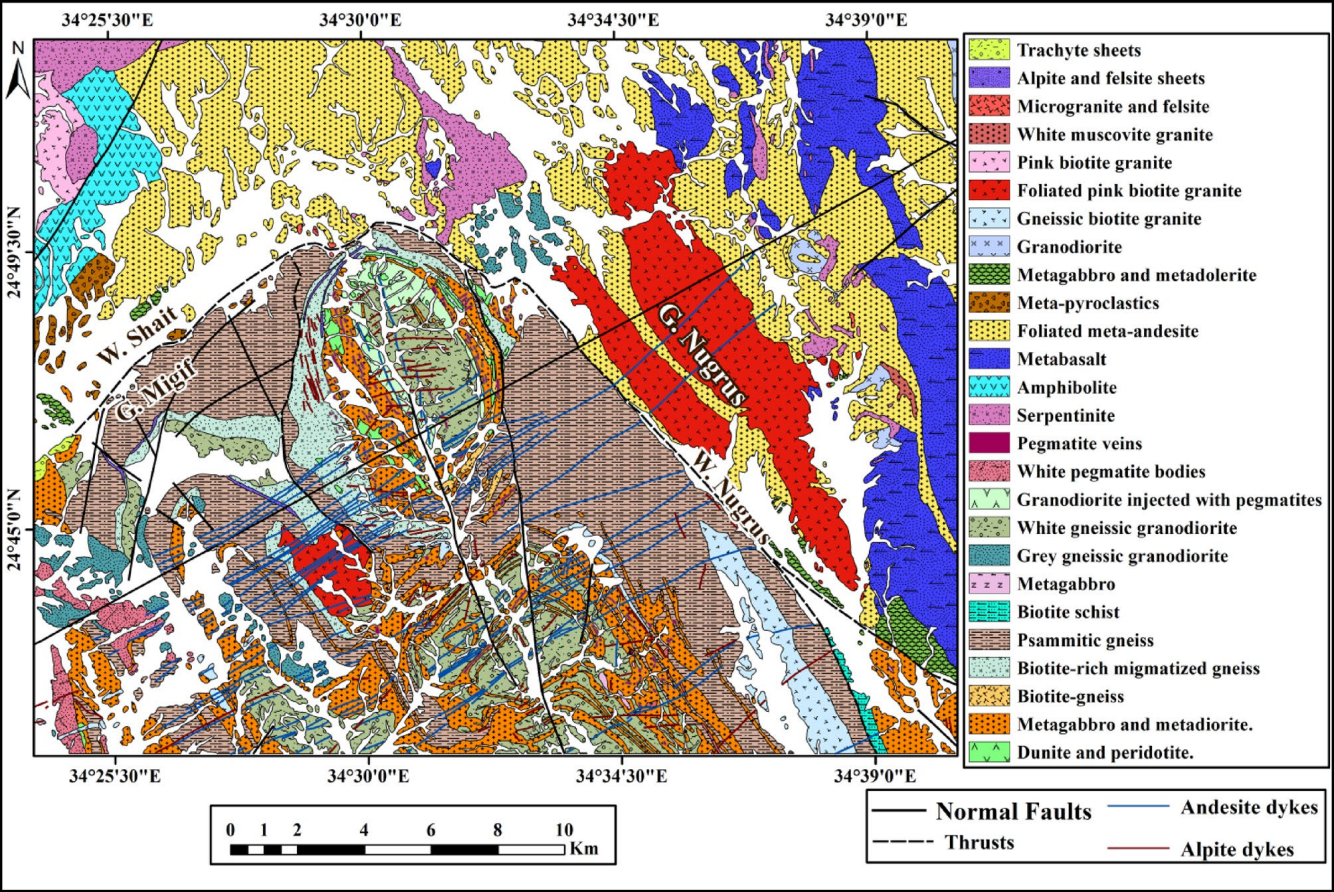
Geological map of the study area modified after^2^. (By ArcGIS v.10.5. https://www.esri.com/en-us/arcgis/products/arcgis-desktop/overview )

Based on the integration of remote sensing analysis and field studies, an updated geological map of the Wadi Shait–Wadi Gemal area has been produced (Fig. 10). This revised map provides a more accurate representation of the different rock units within the study area.

### Field work and petrographic investigation

Field observations and petrographic studies in the Wadi Shait–Wadi Gemal area identified three primary types of garnet-bearing metamorphic rocks: garnetiferous schists, garnet-bearing gneisses, and garnet-bearing pegmatites. Each group is characterized by unique mineral compositions, textures, and deformation patterns.

#### Garnet-bearing schists

**Fig. 11:**
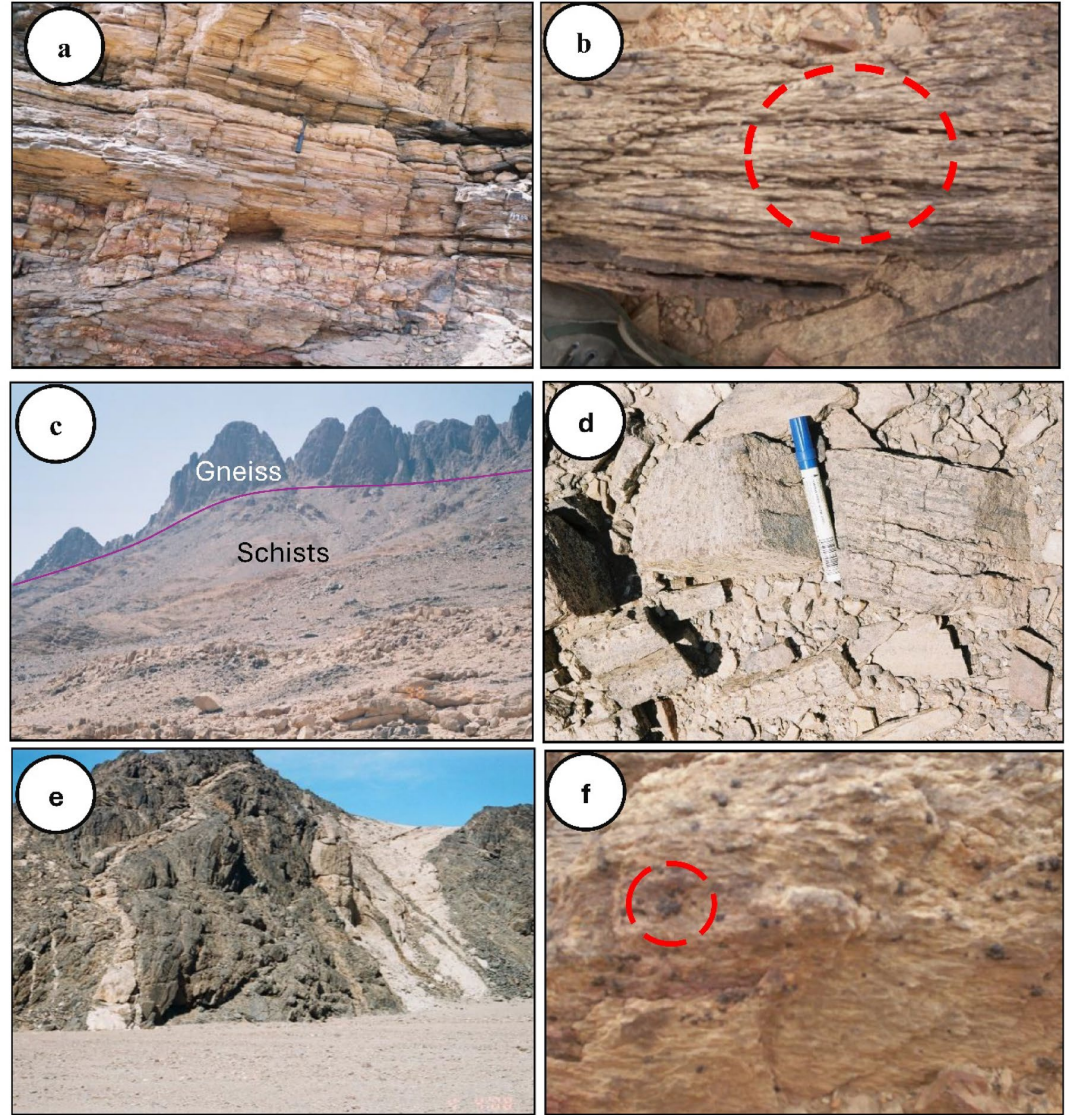
Field observations photographs of the studied rocks. (**a**) well-developed foliation axial planar to asymmetric garnet-bearing schists at Wadi Nugrus. (**b**) close view of garnet minerals-bearing schists at Wadi Nugrus. (**c**) sharp irregular contact between the garnet-bearing gneiss and schists at Wadi Hafafit. (**d**) close-up view of hand-specimen of garnet-gneiss showing garnet large crystals at wadi Nugrus. (**e**) pegmatite veins cut through passamitic gneiss at Wadi Hafafit. **f**) close-up view of hand-specimen of garnet-pegmatite showing garnet large crystals at Wadi Hafafit.

In the Wadi Hafafit and Wadi Nugrus regions, garnet-bearing schists (0.5–1 m thick) are interlayered with mica schists and psammitic gneisses (Fig. 11). These fine-grained, whitish-gray rocks show variable foliation and are mainly composed of quartz and 10–25% biotite, with abundant garnet porphyroblasts (Fig. 11a,b). They formed under amphibolite-facies metamorphism and are believed to originate from sedimentary precursors. Grain sizes range from 0.1 mm to 2.5 cm, and the rocks display features like graded bedding, flaggy structures, and multi-directional joints. Weathering results in polished surfaces, quilted textures, and iron oxide staining.

**Fig. 12:**
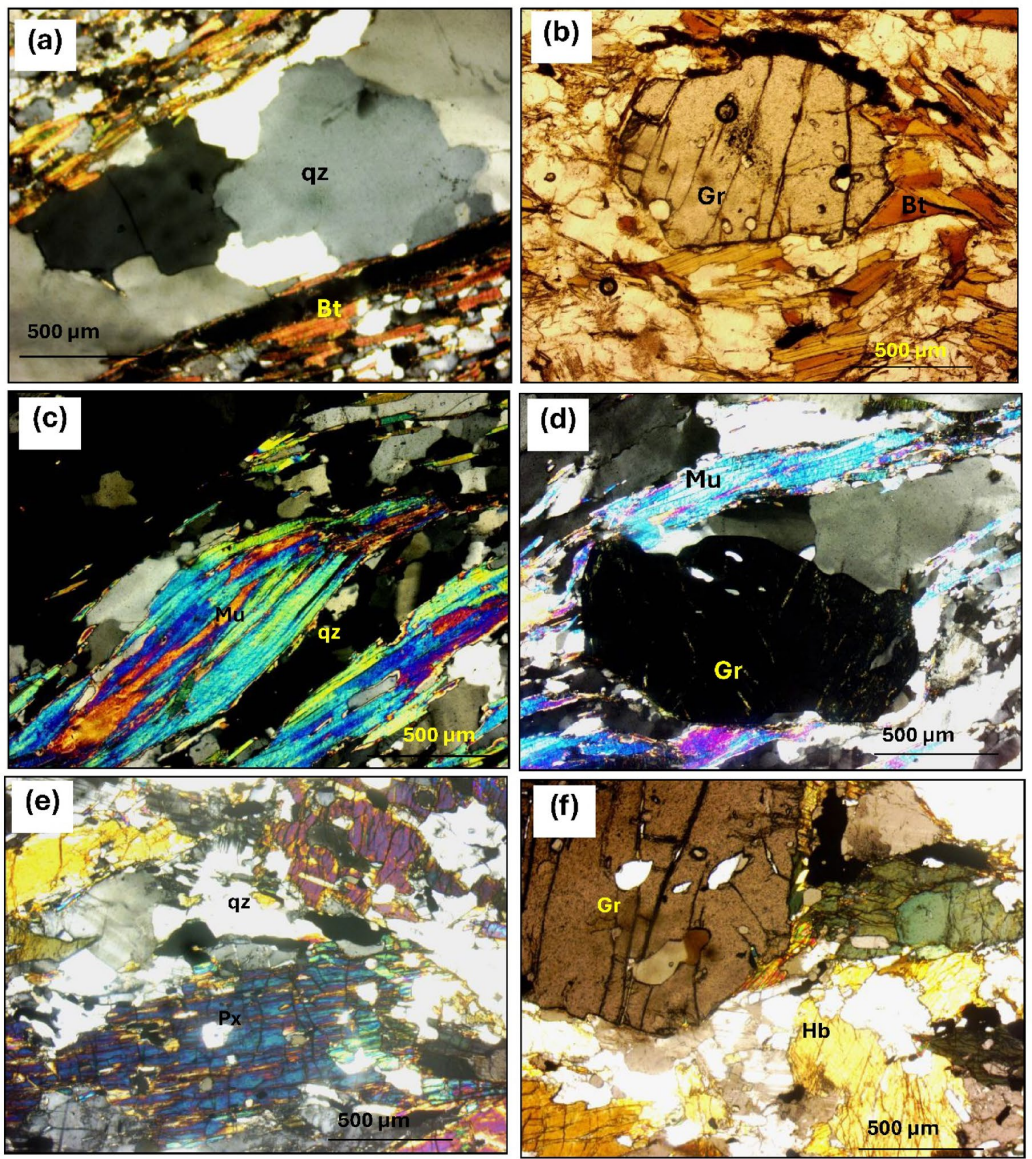
Photomicrographs showing: (**a**) Quartz (Qz) wavy extinction in garnet-biotite schists (C.N.40 X). (**b**) Prismatic biotite (Bi) crystal corroded by groundmass (PPL.). (**c**) Lepidoblastic muscovite (Mu) layer fish-like shape in muscovite-quartz schists (C.N.). (**e**) Garnet (Gr) porphyroblast rounded and enclosing quartz grains (C.N.40 X). **e**) prismatic pyroxene crystals parallel to schistosity planes (C.N.). (**f**) garnet (Gr) idioblastic crystal in hornblende-pyroxene-quartz schist (PPL.40 X).

Four petrographic types are identified:

1. Garnet-biotite-quartz schists composed of quartz, biotite, garnet, and plagioclase, with chlorite as a secondary mineral and iron oxides and apatite as accessories. Quartz appears as subidioblastic to xenoblastic crystals of varying sizes, often strained, fractured, and showing undulose extinction (Fig. 12a). Biotite occurs as elongated, pale brown crystals aligned with schistosity, showing weak pleochroism and sometimes altering to chlorite. Garnet is fractured and includes quartz.
2. Garnet-muscovite-biotite-quartz schists are mainly composed of quartz, biotite, garnet, and muscovite. Quartz appears as coarse, rounded grains showing undulose extinction and signs of pressure-induced segregation. Muscovite is prominent and often intergrown with biotite, while smaller flakes are associated with sericite and epidote. Biotite displays strong pleochroism, ranging from light yellow to dark reddish brown, and often contains pleochroic halos around zircon inclusions. Garnet occurs as both elongated and rounded porphyroblasts (Fig. 12b).
3. Garnet-muscovite-quartz schists is dominated by quartz and muscovite with lepidoblastic textures and kink bands. Quartz appears as xenomorphic to subidiomorphic, show low relief, fracturing, and undulose extinction. Muscovite is abundant, fresh, and unaltered, forming clear white flakes with strong birefringence, sometimes with kink-band deformation (Fig. 12c). Garnet is relatively abundant, occurring as idioblastic, isotropic grains with well-formed crystal shapes, often showing six-sided cross-sections and containing inclusions of quartz and muscovite (Fig. 12d).
4. Hornblende-pyroxene-quartz schist is fine-grained and laminated, composed of quartz, plagioclase, hornblende, augitic pyroxene, and garnet. Hornblende appears as fine- to medium-grained brown crystals with two distinct cleavage planes and strong pleochroism, often showing a dark brown hue. Pyroxene, mainly augite, and occurs as fine-grained, pale yellow to brown crystals, typically randomly oriented and partially to fully altered to chlorite (Fig. 12e). Biotite is present as fine to very fine grains. Garnet forms both elongated and rounded porphyroblasts, often containing inclusions of quartz and muscovite (Fig. 12f).

#### Garnet-bearing gneisses

Garnet-bearing gneisses are the predominant rock type in the gneissic terrains of Hafafit, Nugrus, and Abu Rusheid (Fig. 11c). These dark, resistant rocks exhibit strong foliation and banding, especially in finer-grained varieties, and are mainly composed of garnet and biotite. They often form prominent ridges along the eastern edge of the Hafafit area. At the contact zones between foliated metagabbros and Nugrus granite, garnet-rich quartzo-feldspathic gneisses occur, with garnet replacing biotite as the main mafic mineral. In psammitic layers, garnet content can exceed 20% (Fig. 11d).

**Fig. 13:**
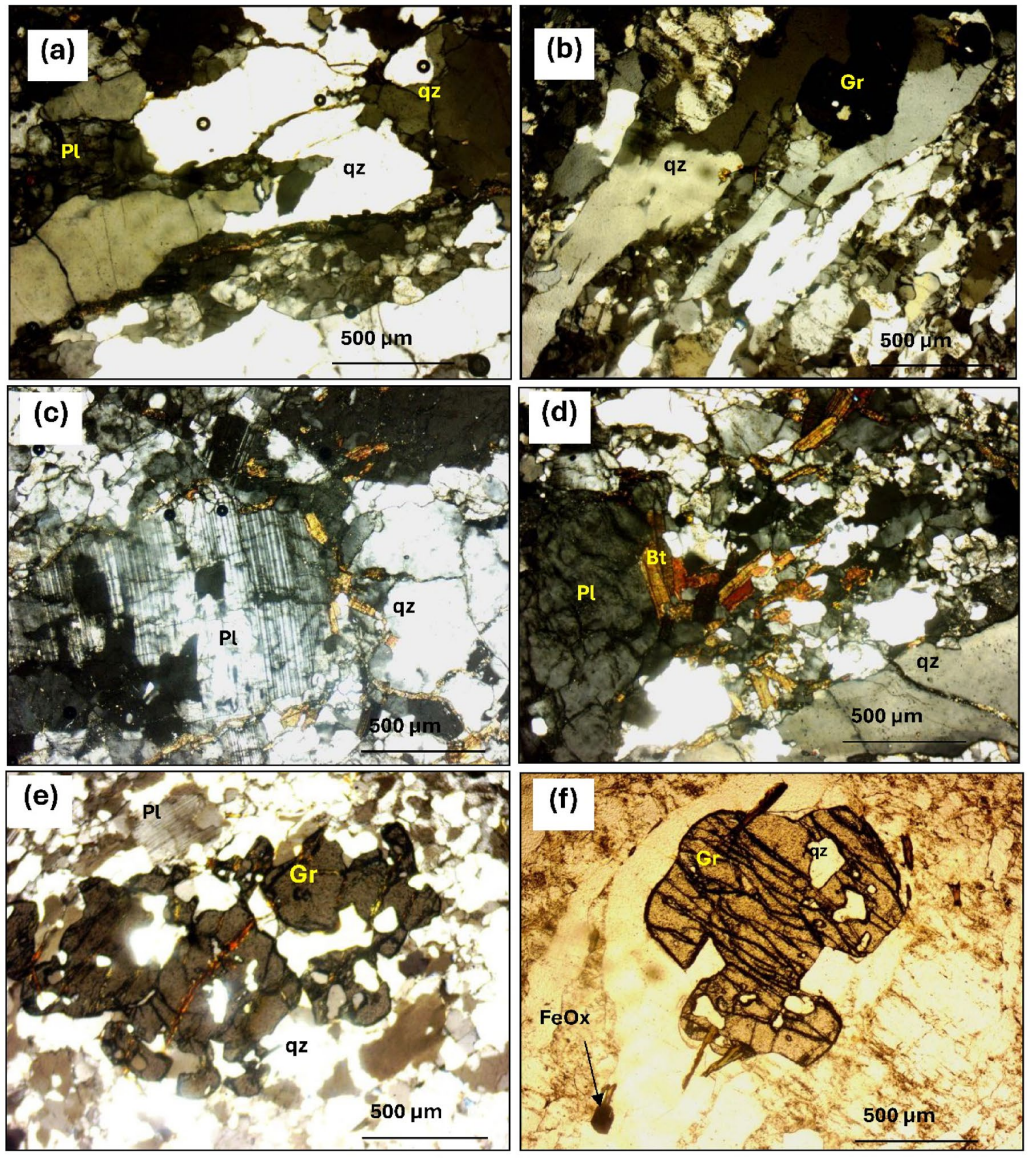
Photomicrographs showing: (**a**) Quartz (qz) deformed crystals in garnet biotite gneiss (C.N.40 X). (**b**) Quartz (qz) ribbons display evidence of grain boundary migration recrystallization, although some grains still exhibit undulatory extinction (C.N.). (**c**) Plagioclase (Pl) cracked crystal shows faint twinning (C.N.40 X). (**d**) Biotite (Bt) aligned flakes in garnet biotite gneiss (PPL.40 X). (**e**) Sigmoidal inclusions trains of quartz in a garnet (Gr) in garnet biotite gneiss (C.N.40 X). (**f**) garnet (Gr) porphyroblast in andesite (PPL.40 X).

Microscopically, these rocks are composed of garnet, biotite, plagioclase, and quartz and have a porphyroblastic texture. Quartz appears as rounded or crushed grains (Fig. 13a), while plagioclase displays zoning and altered twinning (Fig. 13b). Biotite forms pleochroic laths, often altered to chlorite (Fig. 13c,d). Garnet is pink, isotropic, and contains inclusions of quartz and biotite, with larger crystals showing irregular shapes (Fig. 13e,f). Iron oxides are scattered throughout the rock.

#### Garnet-bearing pegmatites

Garnet-bearing pegmatites in the Wadi Hafafit area formed as intrusive veins during the late stages of magmatic differentiation. These veins vary from quartz-feldspar rich to nearly pure quartz, and are typically light-colored with fine- to coarse-grained, saccharoidal textures (Fig. 11e). Their spotted appearance is due to small garnet crystals (up to 0.4 cm; Fig. 11f). Structural features like weak foliation, ptygmatic folds, and boudinage indicate strong tectonic deformation.

**Fig. 14:**
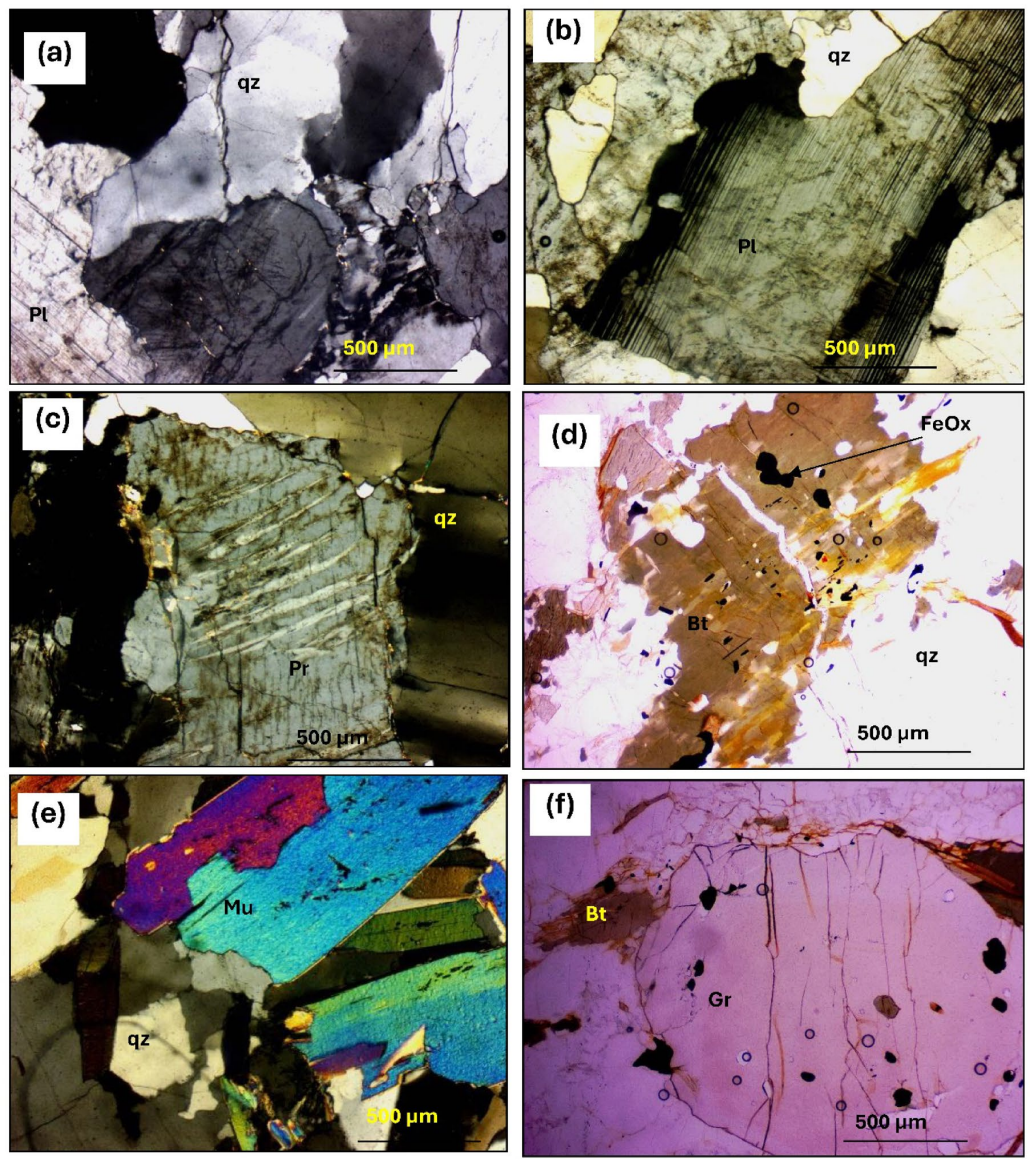
Photomicrographs showing: (**a**) Quartz (Qz) crystal fractured at periphery due to deformation in the garnet bearing pegmatite (C.N.). (**b**) Plagioclase (Pl) with ghost twinning (C.N.40 X). (**c**) Microcline flame perthite (Pr) in the garnet bearing pegmatite (C.N.40 X). (**d**) Biotite (Bt) corroded crystal and encloses iron oxides (FeOx) in the garnet bearing pegmatite (PPL.). (**e**) Muscovite flaky (Mu) crystals the garnet bearing pegmatite (C.N. 40 X). (**f**) Garnet (Gr) crystal porphyroblast in garnet-pegmatite (C.N.).

Petrographically, these pegmatites have composition like alkali feldspar granites, mostly made up of plagioclase, quartz, and microcline, displaying graphic and perthitic textures. Quartz appears as irregular grains intergrown with feldspars (Fig. 14a), while plagioclase (An8–An14) shows deformation and twinning (Fig. 14b). Microcline-perthite exhibits vein, flame, and patchy textures (Fig. 14c). Biotite (about 5%) is pleochroic and often altered (Fig. 14d), while muscovite forms fine laths contributing to early sericitization (Fig. 14e). Garnet, likely almandine, is isotropic and includes quartz, biotite, and iron oxides (Fig. 14f). Apatite is found within biotite, and iron oxides from biotite alteration are the main accessory minerals.

## Discussion

This study demonstrates the value of integrating multi-sensor remote sensing data with field and petrographic investigations to enhance geological mapping in complex terrains. Processing Landsat-9 and ASTER imagery provided a detailed spatial framework that was systematically ground-truthed.

Novel band ratio combinations proved particularly effective for distinguishing garnet-bearing lithologies. For ASTER, the composite (b3/b4, b1/b5, b1/b3) and the ratio (b8/b7) improved the detection of psammitic gneiss. For Landsat-9, the ratios (5/6, 2/4, 5/7) and (5/6, 3/5, 7/6) enabled clear separation of schists, gneisses, and granodiorites. On the processed images, psammitic gneiss appeared violet-blue, biotite-rich gneiss bluish-green, and garnetiferous schists in distinct tones. Applying Principal Component Analysis (PCA) and Minimum Noise Fraction (MNF) transformations further reduced noise and enhanced mineralogical contrasts, resulting in superior lithological discrimination compared to previous mapping efforts.

Fieldwork and petrographic analyses confirmed the correlation between spectral signatures and mineralogical composition. Garnet-bearing schists were identified as fine-grained metamorphic rocks containing garnet porphyroblasts in a quartz-mica matrix. Garnet-rich gneisses exhibited coarse banding and a high garnet content, while pegmatites displayed euhedral garnet crystals within quartz-feldspathic assemblages. These observations validated the spectral interpretations and confirmed the reliability of the proposed workflow.

The integration of remote sensing, field observations, and petrography represents the principal strength of this study. Satellite imagery provided regional coverage, field investigations verified lithological boundaries and structures, and petrographic examination confirmed the mineralogical basis for the spectral responses. This iterative approach reduced interpretation uncertainty and yielded a revised geological map that refines the distribution of garnet-bearing units in the Wadi Shait–Wadi Gemal district.

Although this research focused primarily on lithological discrimination and mapping, the presented workflow also establishes a foundation for future studies on the region’s tectono-metamorphic evolution and offers practical guidance for exploring industrial garnet resources.

## Conclusion

The Wadi Shait–Wadi Gemal district (WSGD), situated within the Migif-Hafafit belt of the Southeastern Desert Tectonic Zone, is a key segment of the Arabian–Nubian Shield that preserves critical records of its tectonic and metamorphic evolution. This study employed an integrated framework combining multi-sensor remote sensing, field mapping, and petrographic investigations to delineate and characterize garnet-bearing lithologies in this structurally complex terrane.

- Multispectral data from Landsat-9 OLI-2 and ASTER sensors were enhanced using various techniques. The proposal band ratio combinations developed for both datasets demonstrated high efficacy in mapping garnetiferous units. ASTER ratios (b3/b4, b1/b5, b1/b3) and (b8/b7) successfully highlighted psammitic gneiss, while Landsat-9 ratios (5/6, 2/4, 5/7) and (5/6, 3/5, 7/6) effectively differentiated garnet-bearing schists, gneisses, and granodiorites.
- Field studies and petrographic investigations confirmed the remote sensing results, revealing three principal garnet-bearing rock types:
  1. Schists, derived from sedimentary protoliths and metamorphosed under amphibolite-facies conditions within thrust-related deformation regimes.
  2. Gneisses, formed during high-grade metamorphism and partial melting associated with Pan-African orogenic crustal thickening.
  3. Pegmatites, enriched in garnet and interpreted as late-stage peraluminous granitic melts emplaced along post-collisional shear zones.

The integration of satellite data with ground validation demonstrates a cost-effective and spatially extensive methodology for refining geological maps and reconstructing Precambrian crustal architecture. Furthermore, the precise identification of garnet-bearing units provides a framework not only for understanding the region’s tectonothermal evolution but also for supporting future economic exploration of garnet, an industrial mineral widely used in abrasives, water-jet cutting, and filtration.

## Data Availability

The datasets generated and analyzed during the current study are available from the corresponding author on reasonable request.
